# The clinical application of longitudinal layer specific strain as a diagnostic and prognostic instrument in ischemic heart diseases: A systematic review and meta-analysis

**DOI:** 10.3389/fcvm.2023.980626

**Published:** 2023-03-27

**Authors:** Shreeya Sharma, Mats Christian Højbjerg Lassen, Anne Bjerg Nielsen, Kristoffer Grundtvig Skaarup, Tor Biering-Sørensen

**Affiliations:** ^1^ Department of Cardiology, Copenhagen University Hospital – Herlev and Gentofte, Copenhagen, Denmark; ^2^ Center for Translational Cardiology and Pragmatic Randomized Trials, Department of Biomedical Sciences, Faculty of Health and Medical Sciences, University of Copenhagen, Copenhagen, Denmark; ^3^ The Copenhagen City Heart Study, Copenhagen University Hospital – Bispebjerg and Frederiksberg, Copenhagen, Denmark

**Keywords:** longitudinal layer specific strain, ischemic heart disease, coronary artery disease, acute coronary syndome, 2 dimensional speckle tracking echocardiography

## Abstract

**Background:**

2-dimensional Speckle-Tracking Echocardiography, to obtain longitudinal layer specific strain (LSS), has recently emerged as a novel and accurate non-invasive imaging technique for diagnosis as well as for prediction of adverse cardiac events. This systematic review and meta-analysis aimed to give an overview of the possible clinical implication and significance of longitudinal LSS.

**Methods:**

We conducted a systematic review and meta-analysis with all the studies involving layer specific strain in patients with ischemic heart disease (IHD). Of 40 eligible studies, 9 met our inclusion criteria. Studies that were included either investigated the prognostic value (*n* = 3) or the diagnostic value (*n* = 6) of longitudinal LSS.

**Results:**

The pooled meta-analysis showed that longitudinal LSS is a significant diagnostic marker for coronary artery disease (CAD) in patients with IHD. Endocardial LSS was found to be a good diagnostic marker for CAD in IHD patients (OR: 1.28, CI95% [1.11–1.48], *p* < 0.001, per 1% decrease). Epicardial (OR: 1.34, CI95% [1.14–1.56], *p* < 0.001, per 1% decrease), Mid-Myocardial (OR: 1.24, CI95% [1.12–1.38], *p* < 0.001, per 1% decrease) and endocardial (OR: 1.21, CI95% [1.09–1.35], *p* < 0.001, per 1% decrease) LSS all entailed diagnostic information regarding CAD, with epicardial LSS emerging as the superior diagnostic marker for CAD in patients with SAP. Endocardial LSS proved to be the better diagnostic marker of CAD in patients with non-ST elevation acute coronary syndrome (NSTE-ACS). LSS was shown to be a good prognostic maker of adverse cardiac events in IHD patients. Two studies found endocardial circumferential strain to be the good predictor of outcome in CAD patients and when added to baseline characteristics. Epicardial LSS emerged as best predictor in acute coronary syndrome (ACS) patients.

**Conclusion:**

In patients with SAP, epicardial LSS was the stronger diagnostic marker while in NSTE-ACS patients, endocardial LSS was the stronger diagnostic marker. In addition, endocardial circumferential strain is the better predictor of adverse outcome in CAD patients whilst in ACS patients, epicardial LSS was found to be a better predictor of outcome.

## Introduction

Despite the extensive and commendable advances in therapeutic treatments, cardiovascular diseases (CVDs) continue to be a leading cause of death worldwide (1), affecting 85.6 million American adults while accounting for approximately one of every three deaths in the United States as of 2016 (2).

Conventionally, cardiac function and contractility is assessed using non-invasive imaging techniques such as echocardiography (1). However, conventional echocardiography is not without limitations, as some CVDs (coronary artery disease) do not necessarily display wall abnormalities detectable by conventional echocardiographic methods (3). Additionally, the conventional methods fail to distinguish the non-homogenous nature of the myocardium with its three layers ranging from endo- to epicardium (4).

With ongoing technological advancements, studies performed during the last decade provide evidence for global longitudinal strain (GLS) obtained from 2-dimensional speckle-tracking echocardiography (2DSTE), as being a robust technique in evaluating left ventricular (LV) systolic function along with being an objective diagnostic marker (5–8). Strain, obtained by 2DSTE is a measure of deformation (5), defined as the percentage change in myocardial segmental length (6).

Novel echocardiographic software can now be used to sectionalize the myocardium in its individual layers allowing for obtainment of the layer specific strain (LSS). This distinction is relevant, especially in ischemic heart disease (IHD), since longitudinally oriented myocardial fibers located in the endocardium region are more susceptible to ischemia (7), as they are located furthest from supplying arteries (8). Longitudinal strain is also impaired amongst patients with subtle cardiac impairment and a preserved left ventricular ejection fraction (LVEF) (9). At the same time, longitudinal layer specific strain (LSS) emerges as a more powerful predictor of outcome than LVEF, as it may reflect even more subclinical LV systolic function (10, 11).

Thus, 2DSTE is likely to contribute further to the pathophysiological and morphological understanding of cardiac diseases (6).

Therefore, the aim of this systematic review and meta-analysis was to evaluate the diagnostic and prognostic value as well as clinical relevance of using 2DSTE measured LSS in patients with suspected or prevalent IHD.

## Methods

### Search process

A trained investigator searched the following databases: PubMed, Embase and Cochrane Library on January 27th 2019. The search strategy included terms and phrases relevant for the subject of the review. The search utilized MeSH terms and free text terms, such as “Layer Specific Strain”, “Speckle Tracking Echocardiography”, “2DSTE”, “Myocardial Strain”, “Global Longitudinal Strain”, “GLS” and “Left ventricular GLS”. Additional search strategies involved reviewing references in the search result to identify further relevant studies to be included. Two investigators (SS and MHL) independently reviewed the results of the searches to determine whether the articles qualified for inclusion in this review.

The search strategy of PubMed is displayed in (Supplementary Table S1).

### Eligibility criteria and study selection

All full text articles describing the prognostic and diagnostic value of 2DSTE measured LSS, in patients with ischemic heart diseases were included in this review. Abstracts, other literature and systematic reviews, conference abstracts, poster presentations, editorials as well as studies reporting results obtained from 3D tracking techniques were excluded from this study.

**Figure 1:**
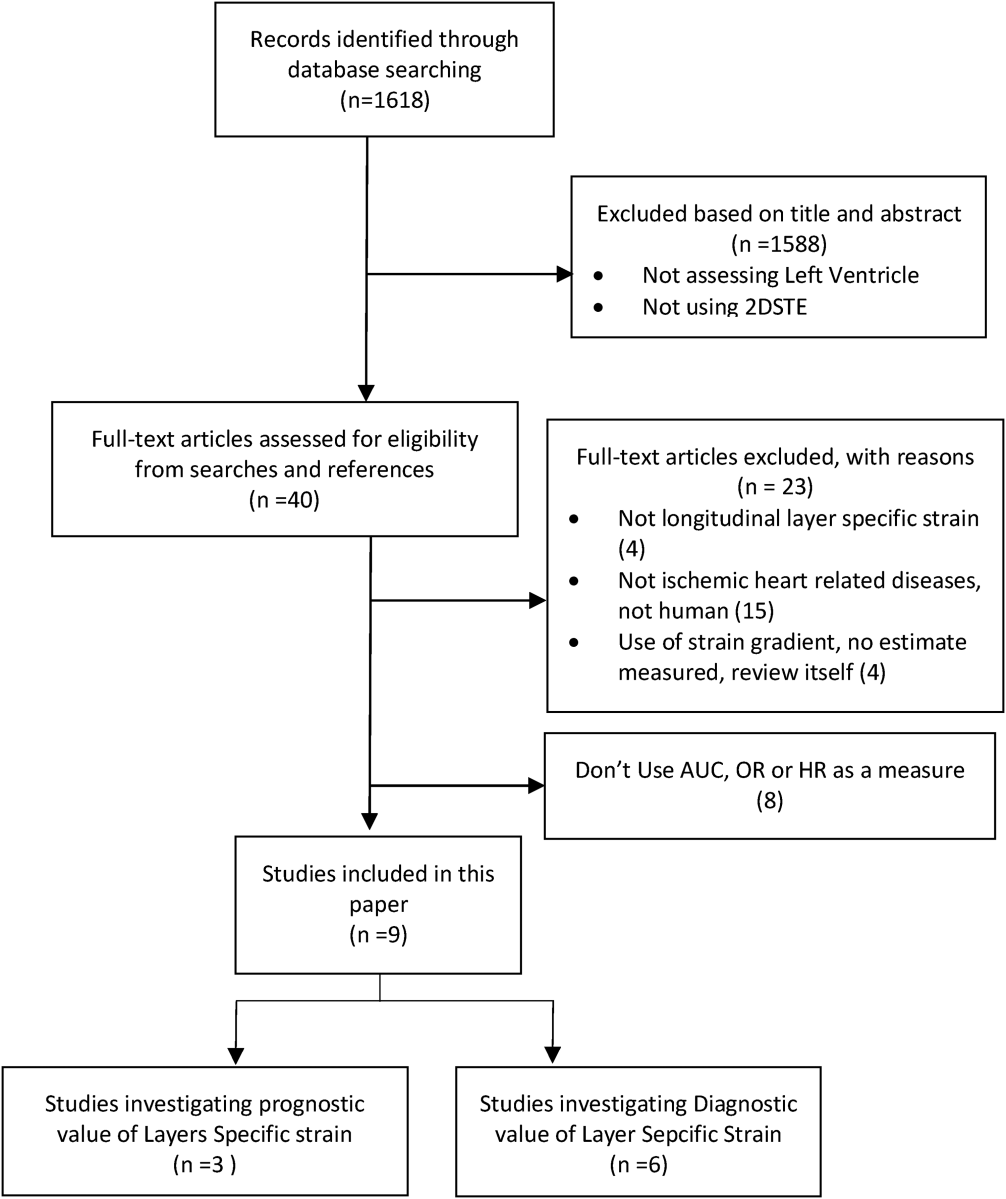
PRISMA flow diagram. PRISMA flow diagram displaying process of finding eligible studies for this review. Abbreviations: PRISMA, preferred reporting items for systematic review and meta-analysis.

Search results were primarily screened based on title and abstract as depicted in the Preferred Reporting Items for Systematic Reviews and Meta-Analyses PRISMA diagram (Figure 1).

From 40 full text articles, 23 were eliminated as the articles were not related to ischemic heart diseases, were reviews, were not LSS or used strain gradient instead of reporting absolute strain values. Furthermore, eight other articles were excluded as they did not use odds ratio (OR) hazard ratio (HR) or area under the curve (AUC) to report their findings.

The qualified studies were divided into two groups; Group 1: studies assessing the diagnostic value (6 studies/Table 1) and Group 2: studies assessing the prognostic value of longitudinal LSS (3 studies/Table 2).

**Table 1:** Studies evaluating the diagnostic significance of LSS.

Author Year	Country	Measurements investigated	Sample size (*n*)	Mean age of CAD pt	EF(%) of CAD pt	Population characteristics	LSS significant as a diagnostic marker for CAD?
Hagemann 2018*	Denmark	Longitudinal LSS (Endo, Epi, Myocardial), CAG	80 (control = 40 with stenosis = 28 without stenosis = 12)	63 ± 11	56 ± 4	Patients with reversible ischemia (SAP)	Yes (Epi-GLS)
Hagemann 2019*	Denmark	Longitudinal LSS (Endo, Epi, Myocardial), CAG, Global Circumferential strain (Endo, epi, myocardial)	285	63.8 ± 10.0	58 ± 4	Patients with SAP	Yes (Epi-GLS)
Yilmaztepe 2018	Turkey	Longitudinal LSS (Endo, Epi, Myocardial), regional longitudinal strains	79	60 ± 9.8	65.4 ± 5.3	Patients with SAP	Yes
Ejlersen, 2017*	Denmark	WM, Longitudinal LSS (Endo, Epi, Myocardial), CAG	132	65.7 Sd (7.2)	63 Sd (10)	Patients with chest pain referred for an invasive coronary angiography	Yes (Epi- GLS)
Sarvari, 2013*	Norway	Territorial longitudinal strain, Longitudinal LSS and Circumferential LSS (for all the cardiac layers)	77 (Coronary occlusion: 28 Stenosis: 21 No stenosis:28)	63.3 ± 9.3	59.0 ± 6	Patients with NSTE-ACS referred to hospital for coronary angiography	Yes (Endo-GLS)
Zhang, 2016	China	Territorial longitudinal strain, Territorial circumferential strain, Longitudinal LSS and Circumferential LSS (for all the cardiac layers)	139	55.4 ± 6.0	61.5 ± 2.0	Patients with NSTE-ACS recommended for undergoing coronary angiography	Yes (Endo-GLS)

* Studies that were included in the meta-analysis.

**Table 2:** LSS as a predictor for adverse cardiac events in IDH patients.

Author Year	Country	Sample size (*n*)	Population characteristics	Length of follow-up	Outcome/Event(s)	Number of events	LSS prognostic value?
Scharrenb-roich 2018	Germany	CAD: 137	CAD and AMI patients	Mean: 3.6 ± 1.2 years	Cardiac death, Hospitalization due to MI, Unstable Angina Pectoris, heart insufficiency	AMI: 22	Yes (Endo-LSS and Endo-GCS)
		AMI: 94				CAD: 47	
Skaarup 2018	Denmark	465	ACS patients	Median: 4.6 (IQR: 0.2–6.3) years	Heart failure or cardiovascular death	199	Yes (epi-LSS)
Hamada 2016	Germany	390	Patients with chronic ischemic cardiomyopathy (defined as known CAD and LVEF ≤50%	Mean: 4.9 ± 2.2 years	Readmission/worsenin-g of heart failure, ventricular arrhythmias, death of any cause.	133	Yes (Endo-GCS)

From Group 1, studies reporting their results as OR were included in this meta-analysis. Three studies (4, 12, 13) reported OR with 95% confidence intervals (CI) for endo-, myo- and epicardium after multivariable adjustments whilst only one of the three studies also reported univariable OR with CI for all three layers. A fourth (5) study also reported OR with CI with both uni- and multivariable adjustments for the endocardial layer only. However, while the first three studies investigated the diagnostic value of LSS in diagnosing CAD in patients with SAP, the fourth study investigated LSS in diagnosing of CAD in NSTE-ACS patients. Pathophysiologically two different conditions are seen and hence the studies were not pooled together. Therefore, three studies were included in this meta-analysis. Because of the heterogeneity amongst the patients in the included studies, a random effect model was utilized. The remaining two studies could not be included in the meta-analysis since they did not report their results with OR. The findings are discussed in the discussion section.

Of the total six studies in the group, five studies (3, 5, 12) provided AUC values for multivariable adjustments whilst only two (3, 5) of the five studies reported the AUCs with 95% CI. Hence, due to lack of sufficient studies reporting AUCs with confidence intervals, AUCs were not included in this meta-analysis.

Group 2 constituted of three studies assessing the prognostic value of LSS in patients with IHD. Out of three studies included in the assessment of prognostic usefulness, one study (14) reported their results as HR whilst the other two studies (15, 16) used AUC. However, the two latter could not be pooled for meta-analysis as the studies used different multivariable adjustment models that could not be pooled together. Due to this, the studies could not be included in the meta-analysis, but their finding were explored in the results and discussions section. While all the studies included in the discussion of prognostic value of LSS focus on longitudinal LSS, two studies (15, 16) also included results of the value of circumferential strain which was also included in this review. Circumferential strain differs from longitudinal strain as it measures the systolic shortening of the short axis of the ventricles while longitudinal strain measures myocardial shortening from base to apex (17).

### Quality assessment

Quality assessment for the risk of bias and the applicability of the included diagnostic studies was evaluated using the Quality Assessment of Diagnostic Accuracy Studies questionnaire (QUADAS-2) (18) (Supplementary Tables S2 and S3). The QUADAS-2 addresses domains regarding the applicability and risk of bias in the studies investigating diagnostic ability. The four domains for risk of bias assessment include patient selection, index test, reference test and flow and timing whilst three domains for applicability include: patient selection, index and reference test. The domains are designated a rating as high, low or moderate risk of bias.

Quality assessment of the studies investigating the prognostic value of LSS were evaluated using the Newcastle–Ottawa Quality Assessment Scale for cohort studies (Supplementary Table S4). This questionnaire consists of three categories: selection, comparability and outcome.

### Statistics

The meta-analysis was conducted using STATA statistics/data analysis, SE 15.0 (StataCorp, College Station, TX, United States). The pooled analyses were performed by using OR extracted from the included studies.

**Figure 2:**
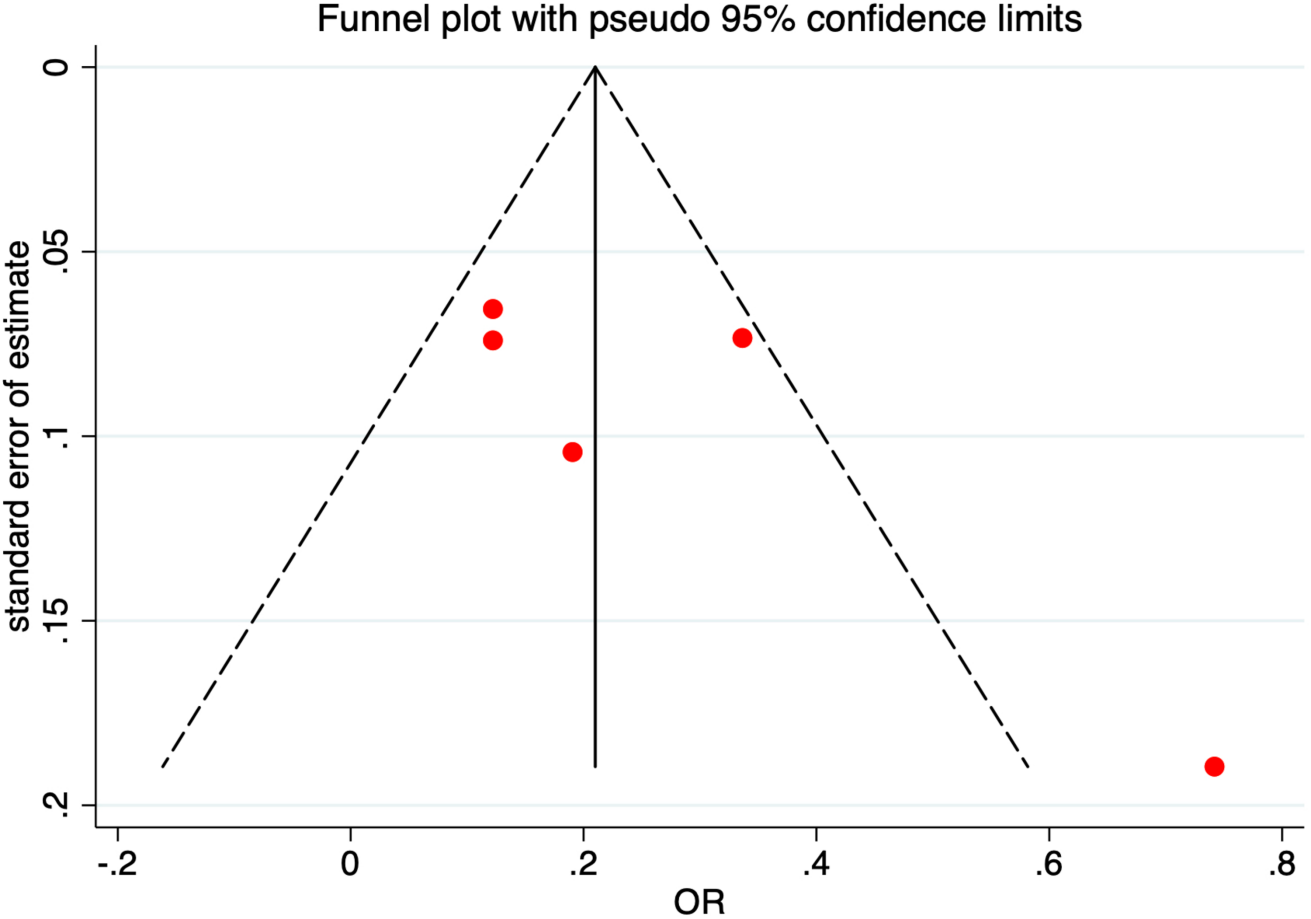
Funnel plot for odds ratio. Funnel plot assessing the possibility of publication bias with pseudo 95% confidence intervals. Abbreviations: SE, standard error; OR, odds ratio.

The results are presented as forest plots. A two-tailed *p*-value of  < 0.05 was defined as statistical significance. The *I*^2^ index was used to assess heterogeneity between studies. All pooled analyses were performed using a fixed effects model and, if heterogeneity was observed (defined as *I*^2^ > 50% or Chi^2^ *p*-value  < 0.10), a random effects model was deployed instead. The possibility of publication bias was assessed using the Egger's test and by visual inspection of funnel plots (Figure 2).

## Results

### Patient characteristics and study design for the studies assessing usefulness of LSS for diagnosing CAD in patients suspected of IHD and predicting outcome in IHD

A total of four studies were included in group 1 of the meta-analysis (*n* = 574). The patient population used in the study is displayed in Table 1. All the patients included underwent echocardiography and CAG in order to diagnose for CAD and confirm the diagnosis of IHD. Some patients (13) also underwent exercise test (*n* = 80) and some underwent SPECT (*n* = 285). Baseline clinical and echocardiographic characteristics of the patients with and without CAD are displayed in Table 3. There were no significant differences between patients with and without CAD in clinical characteristics and comorbidities (all *p*-values > 0.05). There was no significant difference in the LVEF of patients with and without CAD (*p*-value: 0.365). However, GLS endo-, GLS epi-, and GLS mid-myocardium were significantly lower in patients with CAD.

**Table 3:** Baseline characteristics of patients included in the diagnostic studies (group 1).

	Patients without CAD (*n* = 400)	Patients with CAD (*n* = 360)	*P*-value
**General characteristics**
Age	59.1 (53.6–63)	61.9 (55.4–65.7)	0.29
Gender (% male)	52.4 (33.3–79)	76.4 (55–89)	0.12
Smoking (%)	26.3 (19–34)	25.5 (20–36)	0.22
BMI (kg/m^2^)	23 (26–29.7)	27.9 (26.9–29)	0.66
**Comorbidities**
Hypertension (%)	60.3 (52–88.9)	69.9 (46–88.4)	0.42
Diabetes (%)	22.8 (10–27.8)	29.7 (15–62)	0.27
Hypercholestrolemia/Dyslipidemia (%)	46.9 (29–69)	57.5 (37–83)	0.34
Family History (%)	34 (21–49)	38.5 (22–60)	0.66
**Echocardiographic Characteristics**
LVEF (%)	62.2 (57–66.4)	60 (56–65.4)	0.37
GLS endo (%)	22.9 (19.2–28.5)	19.6 (15.4–23.7)	0.0006
GLS epi (%)	17.6 (13.9–21.9)	15.2 (12–16.7)	0.003
GLS mid (%)	19.5 (15.9–18.6)	17.3 (14.9–20.8)	0.0005

Data are expressed as means of the characterisitcs as reported in the studies. The range of means across the studies is reported in (). *P*-value is expressed as the mean *p*-values for studies reporting this value. Abbreviations: LVEF, left ventricular ejection fraction.

The total amount of patients included in group 2 of the meta-analysis included 1,086 patients for who the studies investigated the prognostic value of LSS, and amongst these 401 patients had a cardiac event during the follow-up period. The average follow-up period was 4.3 (0.2–7.1) years. The follow up period along with study characteristics for each study can be seen in Table 2. All the patients involved in the prognostic studies underwent echocardiography. Out of these, some also underwent CAG (*n* = 696) while others were additionally examined using cardiac magnetic resonance imaging (*n* = 390). Baseline clinical and echocardiographic characteristics of: the entire patient population, patients that experienced cardiac event and patients without and cardiac event were compared as displayed in Table 4. All the clinical and echocardiographic characteristics were without any significant difference within the patient groups across the different studies.

**Table 4:** Baseline characteristics of patients included in the prognostic studies (group 2).

	All patients (*n* = 1086)	Cardiac event (*n* = 401)	No Cardiac event (*n* = 685)
**General characteristics**
Age	64 (63–66) [0.035]	68 (67–69) [0.010]	62 (60–64) [0.010]
Gender (% male)	76 (69–85) [0.691]	75.3 (74–84) [0.573]	76.3 (69–86) [0.800]
Smoking (%)	43.5 (39–46) [0.387]	40.4 (37–47.2) [0.453]	44.4 (40–48) [0.430]
SBP (mmHg)	129 (118–137) [0.227]	132.5 (131–134) [0.130]	134.5 (138–131) [0.130]
DBP (mmHg)	76 (72–81) [0.215]	76.5 (73–80) [0.215]	77 (72–82) [0.215]
Heart rate (beats/min)	72 (68–75) [0.562]	73.5 (68–79) [0.446]	69 (67–71) [0.450]
**Comorbidities**
Hypertension (%)	44.2 (41–48.5) [0.282]	43.3 (40–47.5) [0.423]	44.9 (42–49) [0.233]
Diabetes (%)	18.7 (9.7–25) [0.051]	29 (12.1–46) [0.062]	14.1 (7.9–23) [0.050]
Hypercholestrolemia/Dyslipidemia (%)	31.7 (24–39) [0.492]	33.7 (25.1–28) [0.553]	30.6 (23.7–39) [0.494]
Family History (%)	27.8 (25.5–29.9) [0.251]	28.2 (23.6–33) [0.400]	28 (21.5–34.6) [0.367]
**Echocardiographic Characteristics**
LVEF (%)	43.6 (40.8–49) [0.110]	59.9 (35.2–46.5) [0.046]	46.5 (43–51.5) [0.046]
GLS endo (%)	16.1 (14.8–17.5) [0.048]	13.7 (12.5–15) [0.018]	17.9 (16.6–19) [0.018]
GLS epi (%)	11.5 (11–12.5) [0.336]	9.8 (9.3–10) [0.280]	12.7 (12–13.5) [0.280]
GLS total (%)	14.4 (12.8–16) [0.027]	11.4 (10.7–12) [0.033]	15.8 (14.4–17) [0.033]

Data are expressed as means of the characterisitcs as reported in the studies. The range of means across the studies is reported in (). *P*-values expressed as the mean *p*-values for studies reporting this value and reported in []. Abbreviations: SBP, systolic blood pressure; DBP, diastolic blood pressure; LVEF, left ventricular ejection fraction.

**Figure 3:**
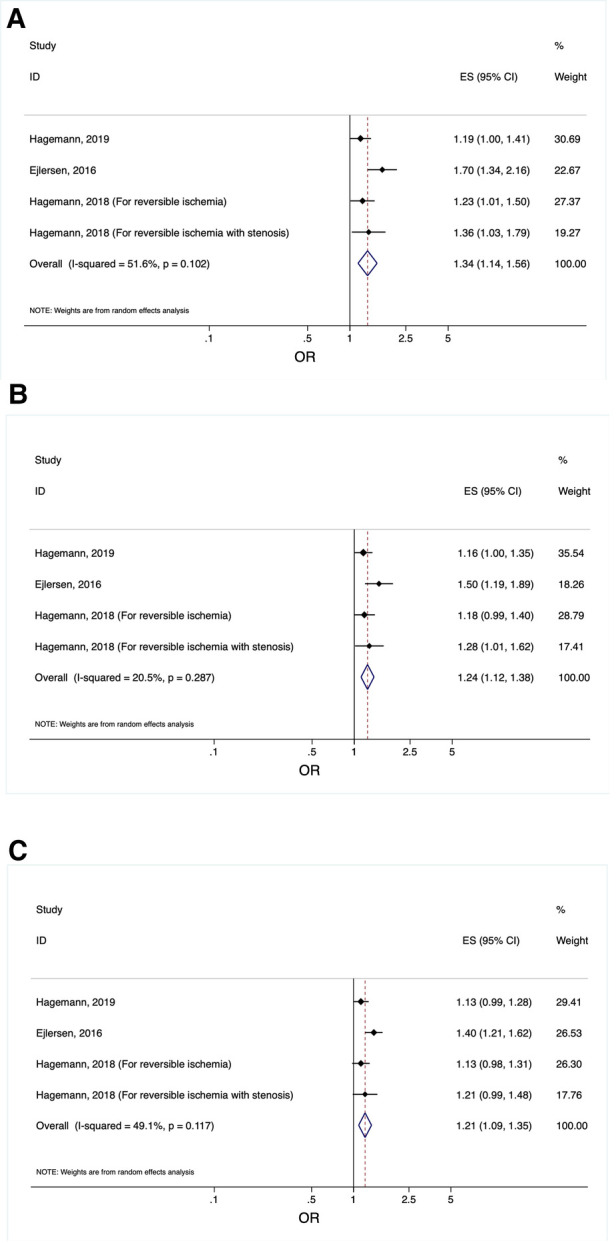
(**A**) Forest plot displaying the odds ratios obtained from multivariable logistic regression models in the included studies investigating the diagnostic value of epicardial longitudinal layer-specific strain in patients suspected of SAP. Odds ratios from the studies investigating the diagnostic value of measuring epicardial longitudinal layer-specific strain in patients suspected of SAP. Abbreviations: OR, odds ratio; CI, confidence interval. (**B**) Forest plot displaying the odds ratios obtained from multivariable logistic regression models in the included studies investigating the diagnostic value of mid-myocardial longitudinal layer-specific strain in patients suspected of SAP. Odds ratios from the studies investigating the diagnostic value of measuring mid-myocardial longitudinal layer-specific strain in patients suspected of SAP. Abbreviations: OR, odds ratio; CI, confidence interval. (**C**) forest plot displaying the odds ratios obtained from multivariable logistic regression models in the included studies investigating the diagnostic value of endocardial longitudinal layer-specific strain in patients suspected of SAP. Odds ratios from the studies investigating the diagnostic value of measuring endocardial longitudinal layer-specific strain in patients suspected of SAP. Abbreviations: OR, odds ratio; CI, confidence interval.

Three studies investigated the diagnostic usability of epi-, mid-myo- and endocardial LSS for coronary artery disease (CAD) in patients suspected of stable angina pectoris (SAP). Hence, three forest plots (one for each myocardial layer) were produced displaying the OR obtained from multivariable logistic regression models in these studies (Figures 3A–C).

### Usefulness of layer specific strain to diagnose coronary artery disease in SAP patients

Three studies investigated the diagnostic value of layer specific strain using OR with a total of 497 patients. From the pooled analysis, it was found that LSS, for all the myocardial layers, were significantly impaired in patients with SAP (Figure 3).

The strongest diagnostic association between LSS and CAD was for epicardial LSS after multivariable adjustment (OR: 1.34, CI95% [1.14–1.56], *p* < 0.001, per 1% decrease) (Figure 3A). Mid-myocardial LSS after multivariable adjustment (OR: 1.24, CI95% [1.12–1.38], *p* < 0.001, per 1% decrease) (Figure 3B) emerged to be the second-best diagnostic marker of CAD in all the myocardial layers. The performance of endocardial LSS as a diagnostic marker for CAD, after multivariable adjustment (OR: 1.21, CI95% [1.09–1.35], *p* < 0.001, per 1% decrease) (Figure 3C), was the weakest of all of the myocardial layers.

### Usefulness of layer specific strain to predict outcome in IHD

Endocardial LSS (HR: 1.19 [1.10–1.28], *p* < 0.001, per 1% decrease) and epicardial LSS (HR: 1.26 [1.15–1.39], *p* < 0.001, per 1% decrease) both prove to provide the most prognostic information about cardiac outcome, with epicardial LSS being the better predictor in ACS patients. In chronic CAD patients, endocardial circumferential strain improves prediction of cardiac event. However, endocardial circumferential strain did not improve prediction of cardiac events in AMI patients (15). In patients with chronic ischemic cardiomyopathy (CAD patients with ejection fraction ≤50%), endocardial circumferential strain (AUC: 0.798, CI95% [0.737–0.833], *p*-value < 0.001) was the strongest prognostic measure followed by endocardial LSS (AUC: 0.780, CI95% [0.706–0.824], *p*-value < 0.001).

There was no evidence of publication bias as evaluated by the Egger's test and from visual inspection of the funnel plot (Figure 2) for the studies assessing LSS as a diagnostic measure for IHDs.

Qualitative assessment of the included studies in the meta-analysis as per QUADAS-2 assessment showed an overall low risk of bias (Supplementary Tables S2 and S3).

## Discussion

The present meta-analysis demonstrates that LSS has significant value as a diagnostic marker for IHD. In patients with SAP, epicardial LSS seems to be the better diagnostic marker for CAD whilst endocardial LSS seems to be the weakest diagnostic marker for CAD. In NSTE-ACS patients, endocardial LSS was the better diagnostic marker for CAD.

In terms of the usefulness of LSS in predicting adverse outcome in IHD, endocardial circumferential strain appears to be a good predictor of adverse outcomes in chronic CAD patients while in ACS patients, epicardial LSS has been found to be a better predictor of outcome.

### Layer-specific strain as a diagnostic marker of CAD

LSS as a new diagnostic tool for IHD has been investigated in a range of patient populations with different cardiac morbidities relating to IHD. Two studies focused on patients with ACS. Sarvari et al. (5) conducted a study aiming to evaluate the use of LSS as a diagnostic marker for CAD, in patients with NSTE-ACS (*n* = 77). Coronary angiography (CAG) was used to confirm CAD in NSTE-ACS patients and found coronary occlusion (*n* = 28), significant stenosis (*n* = 21) and no stenosis (*n* = 28) in patients. Multivariable regression analysis showed that reduced myocardial function as quantified by endocardial LSS was the only significant marker for the presence of significant CAD (OR: 2.10 CI95% [1.47–3.09], *p*-value < 0.001, per 1% change).

Zhang (19) and colleagues carried out a study similar to the study by Sarvari et al., as both studies looked at the usefulness of LSS in diagnosing CAD in patients with NSTE-ACS (*n* = 139). However, Zhang et al. not only looked at the LSS, but also compared its usefulness to the Syntax scoring method. The Syntax scoring method is used to assess the severity of coronary lesions (19). The group of patients with identified CAD were divided into three subgroups (according to the Syntax score). The results were, however, not reported in the form of OR for the LSS for the cardiac layers. Despite not being included in the pooled analysis, the findings of the study mirrored that of Sarvari's study, as endocardial LSS had the best diagnostic value in diagnosing CAD as compared to the other layers. Zhang used coronary angiography to confirm CAD diagnosis in patients.

Four studies focused on evaluating the diagnostic power of LSS in patients with stable angina pectoris (SAP). Two of these studies were published by Hagemann et al. The first study (Hagemann et al., 2018 (13)) was a retrospective study (*n* = 80) in which the objective was to determine whether LSS was affected at rest in patients with reversible ischemia as assessed by single photon emission computed tomography (SPECT) in SAP patients. The control group consisted of 40 patients whilst the study group (*n* = 40) compromised of patients demonstrating stress induced (bicycling or pharmacology stress) reversible ischemia as measured by SPECT. This group was further divided into patients with (*n* = 28) and without (*n* = 12) CAD as defined by a significant stenosis as assessed by CAG (true positive and false positive SPECT). After multivariable logistic regression, the LLS and the severity of reversible ischemia were found to be correlated such that, progressively impaired LSS was observed with no affected major coronary arteries to multivessel ischemia. This association was observed in both true positive and false positive SPECT. The study found that epicardial LSS had the strongest association with ischemia (OR: 1.23, CI95% [1.01–1.50], *p*-value = 0.044). However, the results failed to show a significant association between LSS and the SPECT-measured severity of stenosis.

The works of Ejlersen et al. (12) mirrored the study mentioned above. Ejlersen also evaluated whether LSS under adenosine stress echocardiography provided incremental diagnostic information as compared to traditional echocardiographic measurements with regards to CAD in patients with suspected SAP (*n* = 132). The findings Ejlersen et al. put forward demonstrated that although all three layers were significantly associated with presence of CAD, the epicardium had the highest OR in logistic regressions in multivariable models (OR:1.7 CI95% [1.3–2.1], *p*-value < 0.0001) compared to mid-myocardial LSS (OR:1.5 CI95% [1.3–1.8], *p*-value <0.0001) and endocardial LSS (OR:1.4 CI95% [1.2–1.6], *p*-value < 0.0001).

The second study included by Hagemann et al. (2019) (4) was a prospective study evaluating the potential of LSS for diagnosing CAD in a population of patients with suspected SAP (*n* = 285). All the patients included in the study were examined by echocardiography and an exercise test followed by coronary angiography (CAG). Out of the 285 patients suspected of SAP, 104 had significant CAD whilst 181 had non-significant or no CAD. The study concluded that epi-, mid-myo-, and endocardial LSS were significantly impaired in CAD patients but only epi- and mid-myocardial LSS were independently associated with the presence of significant CAD (epi: OR:1.19, CI95% [1.00–1.41], *p*-value = 0.048 and mid-myocardial: OR: 1.16, CI95% [1.00–1.35], *p*-value = 0.047). After multivariable adjustment, endocardial LSS did not remain independently associated with CAD, and epicardial LSS emerged as being the strongest diagnostic marker.

Both of the studies by Hagemann and colleagues together with the study by Ejlersen and colleagues concluded that epicardial LSS had superior diagnostic accuracy for CAD detection as compared to the mid-myocardial and endocardial LSS in patients suspected of SAP. When considering the cardiac vascular distribution, endocardial layer is considered to be most prone to ischemia is IHD (8) and hence endocardial contractility and in turn endocardial longitudinal strain would be more likely reduced and hence a better diagnostic predictor. However, a possible reason for why epicardial LSS emerged as a superior diagnostic parameter in some studies may be due to technical aspects as the epicardial layer may have more accurate tracing compared to the endocardial region (4). Hagemann et al. And Ejlersen et el. results are in contrast with the findings of Sarvari et al. and Zhang et al. as they found endocardial LSS to have the better diagnostic accuracy in NSTE-ACS patients. The discrepancy in these results can be attributed to the fact that Hagemann et al.'s patient population (suspected SAP) was different from the population used in the two aforementioned studies (NSTEMI).

An additional retrospective study by Yilmaztepe et al. (3) also sought to investigate the diagnostic accuracy of LSS detecting CAD in patients (*n* = 79) with suspected SAP who had previously undergone diagnostic CAG for SAP. The patients were divided into control group (*n* = 36, no significant CAD) whilst 43 patients constituted the CAD group. Since no OR was reported as part of their results, this study was not included in the pooled analysis. However, in a multivariable adjusted model, GLS (AUC: 0.891, CI95% [0.823–0.954], *p*-value < 0.001) along with endocardial LSS (AUC: 0.881, CI95% [0.808–0.905], *p*-value < 0.001) remained independently associated with CAD. These results are different from Hagemann's studies of epicardial GLS being a superior diagnostic marker.

A meta-analysis and systematic review conducted by Liou et al. (20) (10 studies included in analysis) investigated if GLS could be used to improve diagnosis of CAD in patients with SAP or NSTE-ACS. It did, however, not include layer specific GLS. The study found GLS to be a good diagnostic marker for moderate to severe CAD in these patient groups. However, it should be noted that patient groups in these studies show heterogeneity and more studies in the field are needed to subgroup patients in more homogenous groups based on similar pathologies.

### Prognostic value of LSS

In a retrospective study, Skaarup et al. (14), assessed the prognostic value of LSS in predicting heart failure (HF) and cardiovascular death (CD) following ACS in 465 patients. The primary endpoint was the occurrence of HF and/or CD with a median follow up time of 4.6 years (0.2–6.3). Of the patients included 42.7% suffered HF and/or CD (HF = 176 patients and CAD = 38 patients). It was shown that endo- and epicardial LSS were independently associated with the composite outcome (endocardial LSS: HR: 1.19 [1.10–1.28], and epicardial LSS HR: 1.26 [1.15–1.39], *p* < 0.001, per 1% decrease) whilst no other echocardiographic measure remained independently associated with the outcome. In addition to this Skaarup concluded that epicardial LSS, when added to other clinical and echocardiographic measures (such as LVEF and E/e'), provided incremental prognostic information on the risk of developing the endpoint.

Similarly, a prospective study by Scharrenbroich et al. (15) assessed the prognostic value of LSS, in relation to a composite outcome consisting of cardiac death and hospitalization due to MI, in patients previously diagnosed with AMI (*n* = 94) and CAD (*n* = 137). During the follow up time (mean: 3.6 ± 1.2 years) out of the AMI patient group, 22 experienced a cardiac event. At the same time, 47 patients with CAD experienced a cardiac event. While 2DSTE measured strains proved to be a sensitive tool for predicting events in CAD patients, it failed to provide independent prognostic information on adverse events in AMI patients. At the same time the study found endocardial circumferential strain (GCS) to be the measure that, when added to baseline characteristics and ejection fraction, improved the prediction of cardiac events.

Hamada et al. (16) evaluated the prognostic value of LSS, for readmission of heart failure, ventricular arrhythmias or all-cause mortality in patients with known chronic ischemic cardiomyopathy (*n* = 399) (defined as known CAD and LVEF ≤50%). Over the course of the follow up period (mean: 4.9 ± 2.2 years), 133 cardiac events occurred. Endocardial LSS (AUC: 0.780, CI95% [0.706–0.824], *p*-value < 0.001) was found to be a good predictor of the composite outcome while endocardial circumferential strain (AUC: 0.798, CI95% [0.737–0.833], *p*-value < 0.001) emerged to have the strongest prognostic value. However, it should be noted that patients in this study had chronic ischemic cardiomyopathy and therefore a large amount of these patients had a presence of post-infarction scars that can lead to depressed myocardial contractility and deformation. This was one of the reasons why a pooled analysis in the prognostic group was not performed as this would lead to incorrect prognostic results.

### Limitations

There are several limitations to this study. Not all the included studies provided extractable data required for the pooled analysis, thereby limiting the extend of data available for the meta-analysis. In addition to this, patient populations with varying characteristics were included in the studies since the same inclusion and exclusion criteria were not followed across all studies. We cannot differentiate between patients with ischemic heart disease, coronary heart disease and significant coronary stenosis. However, heterogeneity, if present, was analyzed with random effect models to limit this bias. Patients baseline characteristics across studies were also assessed and no significant differences were seen. Diverse variables were included in the multivariable adjustments conducted across the studies resulting in uncertainty in comparison of the effect sizes. Since not many studies included univariable OR, a pooled analysis of the unadjusted effect sizes could not be conducted, for all the layers, in order to overcome this limitation. Results were reported differently across some of the included studies and hence could not be included in our pooled analysis. Furthermore, the study investigating the diagnostic power of LSS in NSTE-ACS patients only reported OR for endocardial layer. Hence, epi- and mid-myocardial layers could not be looked at while investigating the diagnostic significance of these layers in NSTE-ACS patients.

### Clinical implications

With increasing understanding of 2DSTE and its implementation in the field of cardiology globally, longitudinal LSS is likely to prove itself as an accurate non-invasive technique to diagnose and predict conditions in IHD patients. This is due to the increased sensitivity of 2DSTE as compared to other conventional measures allowing for an early diagnosis. Earlier diagnosis by 2DSTE opens up possibilities for early medical intervention in high-risk patients, which may later aid in avoiding adverse outcomes. At the same time, since much of the cost related to cardiovascular diseases is spent on hospitalization due to cardiovascular events (21), early diagnosis and using 2DSTE as a predictive tool could potentially help lower the healthcare costs within the cardiovascular area. Since using 2DSTE to produce longitudinal LSS is mostly automated, it requires minimal training and increases reproducibility.

## Conclusion

We found that 2DSTE measured LSS of the LV has significant diagnostic and prognostic value in patients with suspected and prevalent IHD. Through the studies included in this meta-analysis, it can be concluded that epicardial LSS seem to be the better diagnostic marker for CAD in patients suspected of SAP. Furthermore, it seems that endocardial LSS is a better diagnostic marker for CAD in NSTE-ACS patients. These finding suggests that the usability of LSS for each layer depends on the specific type of IHD. The prognostic value of longitudinal epicardial LSS was found to be predictive of outcome in ACS patients whereas in chronic CAD patients, it was endocardial circumferential strain that proved to be of better prognostic value.

## Data Availability

The original contributions presented in the study are included in the article/**Supplementary Material**, further inquiries can be directed to the corresponding author.
