# Supplementary material for: The clinical application of longitudinal layer specific strain as a diagnostic and prognostic instrument in ischemic heart diseases: A systematic review and meta-analysis
Source: Front Cardiovasc Med. 2023 Mar 27;10:980626. doi: 10.3389/fcvm.2023.980626 (PMC10083306; doi:10.3389/fcvm.2023.980626)
Supplement: Supplementary file 1 [file Datasheet1.pdf]

The clinical application of Longitudinal Layer specific strain as a diagnostic and prognostic instrument in Ischemic Heart Diseases: A Systematic Review and Meta-Analysis

Shreeya Sharma*MB; Mats Christian Højbjerg Lassen* Δ MB; Anne Bjerg Nielsen*MB;

Tor Biering-Sørensen*† MD, PhD, MPH

* Department of Cardiology, Herlev & Gentofte Hospital, University of Copenhagen, Denmark

Δ Division of Cardiology, University of California, San Francisco UCSF

† Department of Biomedical Sciences, Faculty of Health and Medical Sciences, University of Copenhagen, Denmark

Supplemental tables

Supplemental table S1: Search strategy – MEDLINE…………………………………………… 2

Supplemental table S2: Qualitative assessment of study reporting (QUADAS-2)…………………. 3

Supplemental table S3: Summary of QUADAS-2 Assessment of studies investigating the diagnostic value of Layer Specific Strain…………………………………………………………..……... 4

Supplemental table S4: Quality assessment for the observational study…………………...…..... 5

Table S1: Search strategy – MEDLINE

	Search thread	Results
PubMed<br>Date: January 27th 2020	**#1:** (((((Layer Specific Strain) OR Layer Specific Analysis) OR Layer Specific Global longitudinal strain) OR Layer Specific GLS) OR Layer Specific Myocardial strain/Layer Specific left ventricular strain OR Multilayer Longitudinal Strain)	**13881**
	**#2:** (((Speckle Tracking Echocardiography) OR Echocardiography) OR 2-dimensional Speckle tracking Echocardiography) OR 2D-STE	**176570**
	**#3**: #1 OR #2	**190314**
	**#4:** ((((((Coronary Artery disease) OR Stable angina pectoris) OR Non-ST Segment Elevation) OR Myocarditis) OR Ischemic/ Heart Disease) OR Coronary) OR Cardiovascular	**2660159**
	**#5:** (((Coronary) OR Artery Disease) OR Angina pectoris) OR Heart disease	**2040790**
	**#6:** #4 OR #5	**2874597**
	**#7:** #3 and #6	**165305**
	**#8** (((((Left ventricle) OR ventricle) AND Epicardial) OR Subepicardial) OR Endocardial) OR Subepicardial	**22715**
	**#9:** #8 and #7	**3865**
	Filters:<br>Full text, 1980-2019, Humans, English, Adults above 18.	**1654**

Table S2: Qualitative assessment of study reporting (QUADAS-2)

Domain	Questions	Judgments
**Patient selection**		
1) Risk of bias	*Was a consecutive or random sample of patients enrolled?*	Yes, No, Unclear
	Was a case-control design avoided?	Yes, No, Unclear
	Did the study avoid inappropriate exclusions?	Yes, No, Unclear
	Could the selection of patients have introduced bias?	Low, High, Unclear
2)Applicability	*Is there concern that the included patients do not match the review questions?*	Low, High, Unclear
**Index test**	*Were the index test results interpreted without knowledge*	
1) Risk of Bias	*of the results of the reference standard?*	Yes, No, Unclear
	If a threshold was used, was it pre-specified?	Yes, No, Unclear
	Could the conduct or interpretation of the index test have introduced bias?	Low, High, Unclear
2) Applicability	*Is there concern that the index test, its conduct, or*	
	interpretation differ from the review question?	Low, High, Unclear
**Reference standard**	*Is the reference standard likely to correctly classify the*	
1) Risk of Bias	*target condition?*	Yes, No, Unclear
	Were the reference standard results interpreted?	Yes, No, Unclear
	without knowledge of the results of the index test?	Yes, No, Unclear
	Could the reference standard, its conduct, or its interpretation have introduced bias?	Low, High, Unclear
2) Applicability	*Is there concern that the target condition as defined by the*	
	reference standard does not match the review question?	Low, High, Unclear
**Flow and timing**	*Was there an appropriate interval between index test(s)*	
4) Risk of Bias	*and reference standard?*	Yes, No, Unclear
	Did all patients receive a reference standard?	Yes, No, Unclear
	Did patients receive the same reference standard?	Yes, No, Unclear
	Were all patients included in the analysis?	Yes, No, Unclear
	Could the patient flow have introduced bias?	Low, High, Unclear

Table S3: Summary of QUADAS-2 Assessment of studies investigating the diagnostic value of Layer Specific Strain

Author (year) reference	Risk of bias				Applicability concerns		
	Patients selection	Index test	Reference standard	Flow and timing	Patients selection	Index test	Reference standard
Hagemann, 2019	Low	Low	Low	Low	Low	Low	Low
Sarvari, 2013	Low	Unclear	Unclear	Low	Low	Low	Low
Hagemann, 2018	Low	Low	Low	Low	Low	Low	Low
Ejlersen, 2017	Low	Low	Low	Low	Low	Low	Low
Zhang, 2016	Low	Unclear	Unclear	Low	Low	Low	Unclear
Yilmaztepe, 2018	Unclear	Low	Low	Low	Low	Low	Low

Table S4: Quality assessment for the observational study

First author (year)	Selection component				Comparability	Outcome component			Total score
	Representativeness of the exposed cohort	Selection of the non-exposed cohort	Ascertainment of exposure	Demonstration that outcome of interest was not present at start of study		Assessment of outcome	Was follow-up long enough for outcomes to occur	Adequacy of follow up of cohorts	
Hamada (2016)	Somewhat representative of the average Chronic Ischemic Cardiomyopathy community ☆	Drawn from the same community as the exposed cohort ☆	Secure record ☆	Yes ☆	Study controls for all significant variables between those that met the outcome and those who did not. ☆☆	Record linkage and telephone interviews ☆	Yes ☆	Complete follow-up – all subjects accounted for ☆	9
Skaarup (2018)	Truly representative of the average ACS community ☆	Drawn from the same community as the exposed cohort ☆	Secure record ☆	Yes ☆	Study controls for all significant variables; age, gender, BMI, DM, systolic BP and heart rate, LVMI, LVEF, e', E/e' ☆☆	Record linkage ☆	Yes ☆	Complete follow-up – all subjects accounted for ☆	9
Scharrenbroich (2017)	Somewhat representative of the average AMI and CAD community ☆	Drawn from the same community as the exposed cohort ☆	Secure record ☆	Yes ☆	All significant variables were adjusted; age, sex, hypertension, hyperlipidemia, DM, EF. ☆	Record linkage ☆	Yes ☆	Complete follow up - all subjects accounted for ☆	8
